# Supplementary material for: Histopathologic spectrum of focal liver lesions diagnosed by ultrasound-guided percutaneous liver biopsy and predictors of hepatocellular carcinoma: A single-center experience from Sub-Saharan Africa
Source: PLoS One. 2026 Oct 16;21(10):e0357210. doi: 10.1371/journal.pone.0357210 (PMC13674763; doi:10.1371/journal.pone.0357210)
Supplement: Peer review history

# Peer review: Decision Letter 0

**DOI:** 10.1371/journal.pone.0357210.r001

**Authors:** Ashraf Elbahrawy

2 Sep 2026

Dear Dr. Elias,

Thank you for submitting your manuscript to PLOS One. After careful consideration, we feel that it has merit but does not fully meet PLOS One’s publication criteria as it currently stands. Therefore, we invite you to submit a revised version of the manuscript that addresses the points raised during the review process.

Please submit your revised manuscript by Nov 01 2026 11:59PM. If you will need more time than this to complete your revisions, please reply to this message or contact the journal office at plosone@plos.org. When you're ready to submit your revision, log on to https://www.editorialmanager.com/pone/ and select the 'Submissions Needing Revision' folder to locate your manuscript file.

- A letter that responds to each point raised by the academic editor and reviewer(s). You should upload this letter as a separate file labeled 'Response to Reviewers'.
- A marked-up copy of your manuscript that highlights changes made to the original version. You should upload this as a separate file labeled 'Revised Manuscript with Track Changes'.
- An unmarked version of your revised paper without tracked changes. You should upload this as a separate file labeled 'Manuscript'.

If you would like to make changes to your financial disclosure, please include your updated statement in your cover letter. Guidelines for resubmitting your figure files are available below the reviewer comments at the end of this letter.

If applicable, we recommend that you deposit your laboratory protocols in protocols.io to enhance the reproducibility of your results. Protocols.io assigns your protocol its own identifier (DOI) so that it can be cited independently in the future. For instructions see: https://journals.plos.org/plosone/s/submission-guidelines#loc-laboratory-protocols. Additionally, PLOS One offers an option for publishing peer-reviewed Lab Protocol articles, which describe protocols hosted on protocols.io. Read more information on sharing protocols at https://plos.org/protocols?utm_medium=editorial-email&utm_source=authorletters&utm_campaign=protocols.

As the corresponding author, your ORCID iD is verified in the submission system and will appear in the published article. PLOS supports the use of ORCID, and we encourage all coauthors to register for an ORCID iD and use it as well. Please encourage your coauthors to verify their ORCID iD within the submission system before final acceptance, as unverified ORCID iDs will not appear in the published article. *Only*  the individual author can complete the verification step; PLOS staff *cannot*  verify ORCID iDs on behalf of authors.

We look forward to receiving your revised manuscript.

Kind regards,

Ashraf Elbahrawy

Academic Editor

PLOS One

Journal Requirements:

When submitting your revision, we need you to address these additional requirements.

1. Please ensure that your manuscript meets PLOS ONE's style requirements, including those for file naming. The PLOS ONE style templates can be found at

https://journals.plos.org/plosone/s/file?id=wjVg/PLOSOne_formatting_sample_main_body.pdf and

https://journals.plos.org/plosone/s/file?id=ba62/PLOSOne_formatting_sample_title_authors_affiliations.pdf.

2. Please provide a complete Data Availability Statement in the submission form, ensuring you include all necessary access information or a reason for why you are unable to make your data freely accessible. If your research concerns only data provided within your submission, please write "All data are in the manuscript and/or supporting information files" as your Data Availability Statement.

3. We note you have included a table to which you do not refer in the text of your manuscript. Please ensure that you refer to Table 1 & 2 in your text; if accepted, production will need this reference to link the reader to the Table.

4. If the reviewer comments include a recommendation to cite specific previously published works, please review and evaluate these publications to determine whether they are relevant and should be cited. There is no requirement to cite these works unless the editor has indicated otherwise.

[Note: HTML markup is below. Please do not edit.]

Reviewers' comments:

Reviewer's Responses to Questions

**Comments to the Author**

1. Is the manuscript technically sound, and do the data support the conclusions?

Reviewer #1: Yes

Reviewer #2: Yes

**********

2. Has the statistical analysis been performed appropriately and rigorously?

Reviewer #1: Yes

Reviewer #2: Yes

**********

3. Have the authors made all data underlying the findings in their manuscript fully available?

The PLOS Data policy

Reviewer #1: Yes

Reviewer #2: Yes

**********

4. Is the manuscript presented in an intelligible fashion and written in standard English?

Reviewer #1: Yes

Reviewer #2: Yes

**********

Reviewer #1: 1. Title – it invokes sub–Saharan Africa (SSA) however it is a single center study in Ethiopia. The invocation of SSA is not incorrect however it would be beneficial to comment on population studies? Are they all Ethiopians, or is it perhaps ad admixed population of Ethiopians and other SSA nationalities living in Ethiopia? If mostly Ethiopian, I may suggest the title should reflect that and the SSA inference could be in the text. For example - :”an Ethiopian single centre experience”

2. Introduction/Methods

a. The authors note exclusions but do not indicate if ALL patients underwent cross sectional imaging e.g. CT and/or MR with contrast? Could this be noted.

b. The authors should note that for US guided biopsy the lesion must be echobright – some lesions are not and hence are not US guided biopsy possible. This should be noted in introduction or methods section and highlighted that this population represent echobright lesions.

c. The biopsies were assessed using H&E – I would ask that this be expanded as histology for tumour requires H&E for morphology, but differential staining to confirm HCC, or colorectal metastases for example. This should be noted.

3. Did all patients have a single FLL? Or were some multiple? This is noted as pain was a common feature and it seems unusual for a single lesion unless very large.

4. Was alpha fetoprotein available on each patient? If so, it should be added – this would strengthen the paper. The paper talks of tumour markers – I do not see it added anywhere? In addition, if CEA, Ca19-9 was done or collected this too should be added.

5. The lack of correlation between cirrhosis and findings may be confounded by the actual diagnosis of cirrhosis in the study viz “the appearance of cirrhosis on imaging”. I would caution as cirrhosis is either histologically diagnosed or non-invasively through Fibroscan – even an APRI/FIB-4 added to the paper may be helpful.

Reviewer #2: This manuscript addresses an important and clinically relevant topic. The study has potential value; however, several methodological and reporting issues should be addressed before the manuscript can be considered for publication.

Q1. Were all patients included in the study cases in which the diagnosis remained inconclusive despite cross-sectional imaging (CT and/or MRI)? Please clarify the criteria used to define an inconclusive diagnosis.

Q2. The authors did not provide information regarding the size of the focal liver lesions. Please report the lesion size and assess whether lesion size was significantly associated with the diagnosis of hepatocellular carcinoma (HCC). If applicable, please specify the lesion size threshold associated with HCC.

**********

what does this mean?). If published, this will include your full peer review and any attached files.

If you choose “no”, your identity will remain anonymous but your review may still be made public.

**Do you want your identity to be public for this peer review?**  For information about this choice, including consent withdrawal, please see our Privacy Policy

Reviewer #1: No

Reviewer #2: No

**********

[NOTE: If reviewer comments were submitted as an attachment file, they will be attached to this email and accessible via the submission site. Please log into your account, locate the manuscript record, and check for the action link "View Attachments". If this link does not appear, there are no attachment files.]

To ensure your figures meet our technical requirements, please review our figure guidelines: https://journals.plos.org/plosone/s/figures

You may also use PLOS’s free figure tool, NAAS, to help you prepare publication quality figures: https://journals.plos.org/plosone/s/figures#loc-tools-for-figure-preparation.

NAAS will assess whether your figures meet our technical requirements by comparing each figure against our figure specifications.

# Author comment: Author response to Decision Letter 1

**DOI:** 10.1371/journal.pone.0357210.r002

22 Sep 2026

Response to Reviewers

PONE-D-26-32081

Histopathologic Spectrum of Focal Liver Lesions Diagnosed by Ultrasound-Guided Percutaneous Liver Biopsy and Predictors of Hepatocellular Carcinoma: A Single-Center Experience from Sub-Saharan Africa

Dear Editor and Reviewers;

I hope this letter finds you well. I would like to express our gratitude for taking the time to review our manuscript. Your constructive feedback and comments have been invaluable in improving the quality and clarity of our work.

I would like to address the concerns and comments raised in your review. We have carefully considered each one of them and have made the necessary changes to the manuscript accordingly.

So please find the following comments and responses.

Journal Requirements:

When submitting your revision, we need you to address these additional requirements.

1. Please ensure that your manuscript meets PLOS ONE's style requirements, including those for file naming. The PLOS ONE style templates can be found at

https://journals.plos.org/plosone/s/file?id=wjVg/PLOSOne_formatting_sample_main_body.pdf and

https://journals.plos.org/plosone/s/file?id=ba62/PLOSOne_formatting_sample_title_authors_affiliations.pdf.

- Thank you for your feedback. We have thoroughly reviewed the style and formatting guidelines, and have ensured that the manuscript completely meets these standards.

2. Please provide a complete Data Availability Statement in the submission form, ensuring you include all necessary access information or a reason for why you are unable to make your data freely accessible. If your research concerns only data provided within your submission, please write "All data are in the manuscript and/or supporting information files" as your Data Availability Statement.

- "All data are in the manuscript and/or supporting information files" has been included as the Data Availability Statement in the submission form.

3. We note you have included a table to which you do not refer in the text of your manuscript. Please ensure that you refer to Table 1 & 2 in your text; if accepted, production will need this reference to link the reader to the Table.

- Table 1 and Table 2 are now referred to within the text of the manuscript in accordance with the submission guidelines.

4. If the reviewer comments include a recommendation to cite specific previously published works, please review and evaluate these publications to determine whether they are relevant and should be cited. There is no requirement to cite these works unless the editor has indicated otherwise.

- No recommendation was given by the reviewers to cite any specific articles.

Reviewers' comments:

Reviewer's Responses to Questions

Comments to the Author

1. Is the manuscript technically sound, and do the data support the conclusions?

The manuscript must describe a technically sound piece of scientific research with data that supports the conclusions. Experiments must have been conducted rigorously, with appropriate controls, replication, and sample sizes. The conclusions must be drawn appropriately based on the data presented.

Reviewer #1: Yes

Reviewer #2: Yes

- Thank you for the positive response.

________________________________________

2. Has the statistical analysis been performed appropriately and rigorously?

Reviewer #1: Yes

Reviewer #2: Yes

- Thank you for the positive response.

________________________________________

3. Have the authors made all data underlying the findings in their manuscript fully available?

The PLOS Data policy requires authors to make all data underlying the findings described in their manuscript fully available without restriction, with rare exception (please refer to the Data Availability Statement in the manuscript PDF file). The data should be provided as part of the manuscript or its supporting information, or deposited to a public repository. For example, in addition to summary statistics, the data points behind means, medians and variance measures should be available. If there are restrictions on publicly sharing data—e.g. participant privacy or use of data from a third party—those must be specified.

Reviewer #1: Yes

Reviewer #2: Yes

- Thank you for the positive response.

________________________________________

4. Is the manuscript presented in an intelligible fashion and written in standard English?

PLOS ONE does not copyedit accepted manuscripts, so the language in submitted articles must be clear, correct, and unambiguous. Any typographical or grammatical errors should be corrected at revision, so please note any specific errors here.

Reviewer #1: Yes

Reviewer #2: Yes

- Thank you for the positive response.

________________________________________

5. Review Comments to the Author

Please use the space provided to explain your answers to the questions above. You may also include additional comments for the author, including concerns about dual publication, research ethics, or publication ethics. (Please upload your review as an attachment if it exceeds 20,000 characters)

Reviewer #1:

1. Title – it invokes sub–Saharan Africa (SSA) however it is a single center study in Ethiopia. The invocation of SSA is not incorrect however it would be beneficial to comment on population studies? Are they all Ethiopians, or is it perhaps ad admixed population of Ethiopians and other SSA nationalities living in Ethiopia? If mostly Ethiopian, I may suggest the title should reflect that and the SSA inference could be in the text. For example - :”an Ethiopian single centre experience”

- Thank you for the feedback. While the majority of patients in our study are Ethiopian, a significant portion of our study participants also include nationals from neighboring East African and sub-Saharan African countries such as Somalia, Djibouti, Eritrea, and South Sudan who seek care at Adera Medical & Surgical Center, a major regional tertiary referral hub for gastroenterology and hepatology services. Because our study population naturally reflects this broader regional demographic, we felt the use of "sub-Saharan Africa" in the title was justified. To clarify this for the reader, we have updated the study setting section under Methodology to explicitly state that the institution regularly receives and manages patients from other parts of East Africa.

2. Introduction/Methods

a. The authors note exclusions but do not indicate if ALL patients underwent cross sectional imaging e.g. CT and/or MR with contrast? Could this be noted.

- Thank you for pointing this out. All study participants underwent contrast-enhanced cross-sectional imaging (CT and/or MRI), and the presence of focal liver lesions on these scans was the primary entry criterion for the study. To ensure clarity for the readers, we have revised the Study Population section under Methodology to explicitly state this requirement.

b. The authors should note that for US guided biopsy the lesion must be echobright – some lesions are not and hence are not US guided biopsy possible. This should be noted in introduction or methods section and highlighted that this population represent echobright lesions.

- Thank you for this valuable point. We have revised the "Biopsy Procedure and Histopathologic Evaluation" section within the Methodology to clarify the reasons for lesion inaccessibility. We now specify that lesions were excluded if they were sonographically inaccessible due to challenging anatomical locations or poor visibility on ultrasound, including lesions that were not sufficiently echobright to safely guide a biopsy.

c. The biopsies were assessed using H&E – I would ask that this be expanded as histology for tumour requires H&E for morphology, but differential staining to confirm HCC, or colorectal metastases for example. This should be noted.

- Thank you for this important note. We agree that while H&E staining provides the essential morphological framework, differential staining is critical for definitive tumor classification. In our study, immunohistochemical markers (such as HepPar-1, Glypican-3, CK7, CK20, and CDX2) were utilized by the pathologists when necessary to distinguish primary hepatocellular carcinoma from metastatic lesions or cholangiocarcinoma. We have updated the "Biopsy Procedure and Histopathologic Evaluation" section in the Methodology to explicitly detail the special stains and IHC panel used for definitive diagnosis.

3. Did all patients have a single FLL? Or were some multiple? This is noted as pain was a

common feature and it seems unusual for a single lesion unless very large.

- Thank you for this insightful observation. Our cohort included a significant proportion of patients with multiple lesions; specifically, more than a third of the study participants (38.7%) presented with multifocal focal liver lesions, which accounts for the high prevalence of abdominal pain observed. We have updated the Results section to include this distribution of single versus multifocal lesions to provide better context for the clinical presentation.

4. Was alpha fetoprotein available on each patient? If so, it should be added – this would strengthen the paper. The paper talks of tumour markers – I do not see it added anywhere? In addition, if CEA, Ca19-9 was done or collected this too should be added.

- Thank you for the suggestion. While tumor markers including AFP, CEA, and CA 19-9 were routinely requested as part of the clinical workup for the majority of patients, they were excluded from the final analysis because the tests were performed across three external laboratories utilizing highly varied reference ranges and assay cutoffs. Aggregating this heterogeneous data would have introduced significant confounding variables and reporting biases. We have updated the Limitations section of our Discussion to clarify the omission of these markers.

5. The lack of correlation between cirrhosis and findings may be confounded by the actual diagnosis of cirrhosis in the study viz “the appearance of cirrhosis on imaging”. I would caution as cirrhosis is either histologically diagnosed or non-invasively through Fibroscan – even an APRI/FIB-4 added to the paper may be helpful.

- Thank you for this critical insight. We agree that imaging features alone can underdiagnose or misclassify cirrhosis, which likely confounded our initial analysis. In accordance with your excellent recommendation, we calculated the non-invasive fibrosis scores (FIB-4) for our cohort using baseline laboratory data. Interestingly, while imaging-defined cirrhosis lacked a correlation, the FIB-4 score demonstrated a statistically significant association with the presence of HCC (p = 0.038), with higher scores observed in the HCC group. This clearly demonstrates that evaluating the underlying liver background through non-invasive biochemical markers resolves the confounding limitations of imaging. We have updated the Methods and Results sections and added a discussion on these findings to the manuscript.

Reviewer #2: This manuscript addresses an important and clinically relevant topic. The study has potential value; however, several methodological and reporting issues should be addressed before the manuscript can be considered for publication.

Q1. Were all patients included in the study cases in which the diagnosis remained inconclusive despite cross-sectional imaging (CT and/or MRI)? Please clarify the criteria used to define an inconclusive diagnosis.

- We thank the reviewer for this question. Yes, all patients included in this study represented cases where the diagnosis remained inconclusive or indeterminate despite cross-sectional imaging (CT and/or MRI). Per our protocol, an imaging diagnosis was defined as 'inconclusive' if the focal liver lesion could not be definitively categorized as benign or malignant using standard imaging criteria. Specifically, we utilized the Liver Imaging Reporting and Data System (LI-RADS) to objectively define this threshold. By excluding lesions categorized as LI-RADS 1 or 2 (definitely/probably benign) and LI-RADS 5 (definitely HCC), our final cohort strictly comprised intermediate or indeterminate lesions (LI-RADS 3, 4, and M) that required histopathological confirmation via US-PLB. To make this completely clear for readers, we have added a clarifying phrase to this sentence in the methods section of the revised manuscript.

Q2. The authors did not provide information regarding the size of the focal liver lesions. Please report the lesion size and assess whether lesion size was significantly associated with the diagnosis of hepatocellular carcinoma (HCC). If applicable, please specify the lesion size threshold associated with HCC.

- We thank the reviewer for this constructive suggestion. We have updated the manuscript to include a comprehensive analysis of focal liver lesion size:

• Lesion Size Reporting: The mean lesion size was 8.12 ± 3.89cm, with a median size of 8.20cm (IQR: 5.10 - 11.00; Range: 0.9 - 16.7cm). These baseline descriptive statistics have been integrated into the results section.

• Statistical Association: A Mann-Whitney U test confirmed that larger lesion size was highly significantly associated with an HCC diagnosis (U = 854.0, Z = -3.651, p < 0.001), with a significantly higher size ranking observed in the HCC group (Mean Rank: 66.57 vs. 44.47).

• Diagnostic Threshold: An ROC curve analysis demonstrated acceptable discriminatory power with an AUC of 0.705 (p < 0.001). Maximizing Youden’s Index yielded an optimal clinical threshold of 8.95cm, providing a sensitivity of 63.3% and a specificity of 74.6% for predicting biopsy-confirmed HCC. The Methods and Results sections have been revised to clearly reflect these sizing parameters and diagnostic cutoff value.

Attachment Submitted filename: Response to Reviewers.docx

# Peer review: Decision Letter 1

**DOI:** 10.1371/journal.pone.0357210.r003

**Authors:** Ashraf Elbahrawy

5 Oct 2026

<p>Histopathologic Spectrum of Focal Liver Lesions Diagnosed by Ultrasound-Guided Percutaneous Liver Biopsy and Predictors of Hepatocellular Carcinoma: A Single-Center Experience from Sub-Saharan Africa

PONE-D-26-32081R1

Dear Dr. Elias,

We’re pleased to inform you that your manuscript has been judged scientifically suitable for publication and will be formally accepted for publication once it meets all outstanding technical requirements.

Within one week, you’ll receive an e-mail detailing the required amendments. When these have been addressed, you’ll receive a formal acceptance letter and your manuscript will be scheduled for publication.

An invoice will be generated when your article is formally accepted. Please note, if your institution has a publishing partnership with PLOS and your article meets the relevant criteria, all or part of your publication costs will be covered. Please make sure your user information is up-to-date by logging into Editorial Manager at Editorial Manager® and clicking the ‘Update My Information' link at the top of the page. For questions related to billing, please contact billing support.

If your institution or institutions have a press office, please notify them about your upcoming paper to help maximize its impact. If they’ll be preparing press materials, please inform our press team as soon as possible -- no later than 48 hours after receiving the formal acceptance. Your manuscript will remain under strict press embargo until 2 pm Eastern Time on the date of publication. For more information, please contact onepress@plos.org.

Kind regards,

Ashraf Elbahrawy

Academic Editor

PLOS One

Additional Editor Comments (optional):

Reviewers' comments:

Reviewer's Responses to Questions

**Comments to the Author**

Reviewer #1: All comments have been addressed

Reviewer #2: All comments have been addressed

**********

2. Is the manuscript technically sound, and do the data support the conclusions?

Reviewer #1: Yes

Reviewer #2: Yes

**********

3. Has the statistical analysis been performed appropriately and rigorously?

Reviewer #1: Yes

Reviewer #2: Yes

**********

4. Have the authors made all data underlying the findings in their manuscript fully available?

The PLOS Data policy

Reviewer #1: Yes

Reviewer #2: Yes

**********

5. Is the manuscript presented in an intelligible fashion and written in standard English?

Reviewer #1: Yes

Reviewer #2: Yes

**********

Reviewer #1: Thank you for addressing my question adequately. I still think that adding AFP would be helpful but adding it as a limitation is reasonable. I am pleased the FIB-4 correlates well.

Reviewer #2: (No Response)

**********

what does this mean?). If published, this will include your full peer review and any attached files.

If you choose “no”, your identity will remain anonymous but your review may still be made public.

**Do you want your identity to be public for this peer review?**  For information about this choice, including consent withdrawal, please see our Privacy Policy

Reviewer #1: No

Reviewer #2: No

**********

# Editor report: Acceptance letter

**DOI:** 10.1371/journal.pone.0357210.r004

**Authors:** Ashraf Elbahrawy

PONE-D-26-32081R1

PLOS One

Dear Dr. Elias,

I'm pleased to inform you that your manuscript has been deemed suitable for publication in PLOS One. Congratulations! Your manuscript is now being handed over to our production team.

At this stage, our production department will prepare your paper for publication. This includes ensuring the following:

* All references, tables, and figures are properly cited

* All relevant supporting information is included in the manuscript submission,

* There are no issues that prevent the paper from being properly typeset

You will receive further instructions from the production team, including instructions on how to review your proof when it is ready. Please keep in mind that we are working through a large volume of accepted articles, so please give us a few days to review your paper and let you know the next and final steps.

Lastly, if your institution or institutions have a press office, please let them know about your upcoming paper now to help maximize its impact. If they'll be preparing press materials, please inform our press team within the next 48 hours. Your manuscript will remain under strict press embargo until 2 pm Eastern Time on the date of publication. For more information, please contact onepress@plos.org.

You will receive an invoice from PLOS for your publication fee after your manuscript has reached the completed accept phase. If you receive an email requesting payment before acceptance or for any other service, this may be a phishing scheme. Learn how to identify phishing emails and protect your accounts at https://explore.plos.org/phishing.

If we can help with anything else, please email us at customercare@plos.org.

Thank you for submitting your work to PLOS One and supporting open access.

Kind regards,

PLOS One Editorial Office Staff

on behalf of

Prof. Ashraf Elbahrawy

Academic Editor

PLOS One
